# *KANSL2* and *MBNL3* are regulators of pancreatic ductal adenocarcinoma invasion

**DOI:** 10.1038/s41598-020-58448-y

**Published:** 2020-01-30

**Authors:** Peter O. Oladimeji, Jesse Bakke, William C. Wright, Taosheng Chen

**Affiliations:** 1 Department of Chemical Biology and Therapeutics, St. Jude Children’s Research Hospital, Memphis, Tennessee 38105 USA; 2 Present Address: Department of Foundational Science, Central Michigan University, Mt. Pleasant, Michigan 48859 USA; 3 Integrated Biomedical Sciences Program, University of Tennessee Health Science Center, Memphis, Tennessee 38163 USA

**Keywords:** High-throughput screening, Pancreatic cancer

## Abstract

Pancreatic ductal adenocarcinoma (PDAC) is one of the most lethal forms of cancer. One major reason for this is that PDAC quickly metastasizes to other organs, thereby making its treatment difficult. The molecular machinery driving PDAC metastasis is still poorly understood. In this study, we applied an unbiased approach using CRISPR screening to identify genes that strongly regulate invasion (based on an *in vitro* assessment of their metastatic potential) in PANC-1, a PDAC cell line. Through CRISPR screening, we identified *MBNL3* and *KANSL2* as strong regulators of invasion in PANC-1 cells. We further validated *MBNL3* and *KANSL2* as regulators of PANC-1 cell invasion by using the doxycycline-inducible shRNA system. We also showed that *MBNL3* and *KANSL2* do not affect cell proliferation. Through our efforts, we have established a process to identify genes that regulate cell invasion and can be further investigated as potential targets for therapeutic intervention.

## Introduction

Relapse and metastasis of cancers can occur months, years, or even decades after the treatment of the primary tumor, and this can impose a great burden on patients^1^. Metastases of various cancers cause the vast majority of deaths of cancer patients and pose a formidable challenge to oncologists^2^. When metastasis manifests after the surgical removal of a primary tumor, systemic treatments are often used. These treatments include classical chemotherapy, new targeted therapy, immunotherapy or a combination of these therapeutic approaches^2,3^. Despite all efforts on the research and medical fronts to treat metastatic tumors, current therapies often achieve only partial response of metastatic tumors. Continued treatment may keep the residual tumor quiescent only temporarily; however, from the residual cancer cell population, drug-resistant clones ultimately emerge and lead to rapid relapse^4^. Hence, there are currently no effective therapies for treating metastatic cancer and cure rates for patients with metastases is low.

Pancreatic cancer, one of the most aggressive malignant neoplasms, is currently the fourth leading cause of cancer-related deaths in Western society. However, by 2030, it is predicted to surpass breast, prostate, and colorectal cancer, and become the second leading cause of cancer-related mortality after lung cancer^5^. According to the estimates by American Cancer Society, the number of people to be diagnosed with pancreatic cancer will be approximately 55,440, and approximately 44,330 people will die of the disease in the USA alone in 2018^6^. According to the most recent estimates of the National Cancer Institute’s Surveillance, Epidemiology, and End Results (SEER) program, 52% of all the pancreatic cancers diagnosed between 2007 and 2013 metastasized to distant organs and 29% of them had spread to regional lymph nodes. The 5-year survival rate for all patients with pancreatic cancer during that same period was 8.2% overall for all stages^7^, a rate that is largely a result of the high metastatic potential of pancreatic cancer. Pancreatic ductal adenocarcinoma (PDAC) accounts for 95% of all pancreatic neoplasia^8^ and is characterized by a highly aggressive phenotype that includes invasiveness, proliferation, and resistance to drugs^3,9^. Metastasis of pancreatic cancer remains a major challenge in treating this disease. A better understanding of the molecular mechanism responsible for the development of metastatic pancreatic cancer will facilitate the development of new therapy; therefore, it is important to identify important mediators of pancreatic cancer metastasis.

In this study, we present for the first time an unbiased approach to identifying genes that regulate PDAC cell metastasis as assessed by cell invasive capacity. By using a genome-scale clustered regularly interspaced short palindromic repeats (CRISPR) approach, coupled with follow-up validation using both CRISPR knockout and small interfering RNA (siRNA) knockdown, we identified and validated *KANSL2* and *MBNL3* as regulators of PDAC invasion.

## Results

### A CRISPR/Cas9 screen identifies modulators of PDAC cell invasion

**Figure 1:**
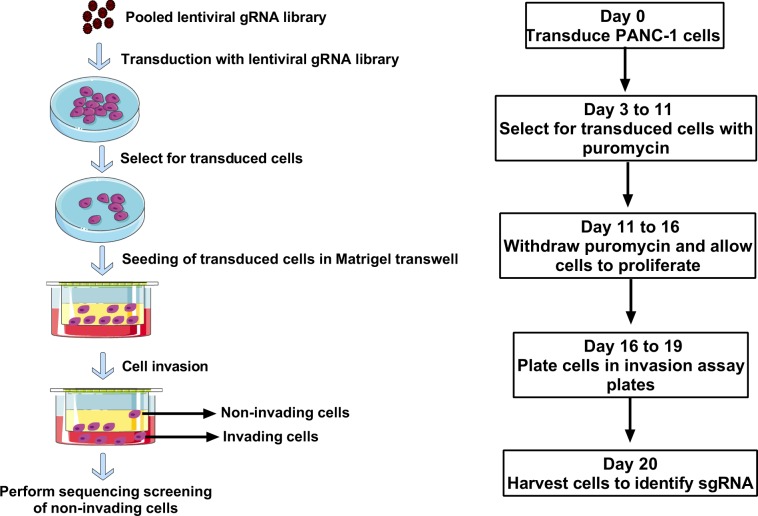
Schematic overview of the CRISPR screening workflow.

**Figure 2:**
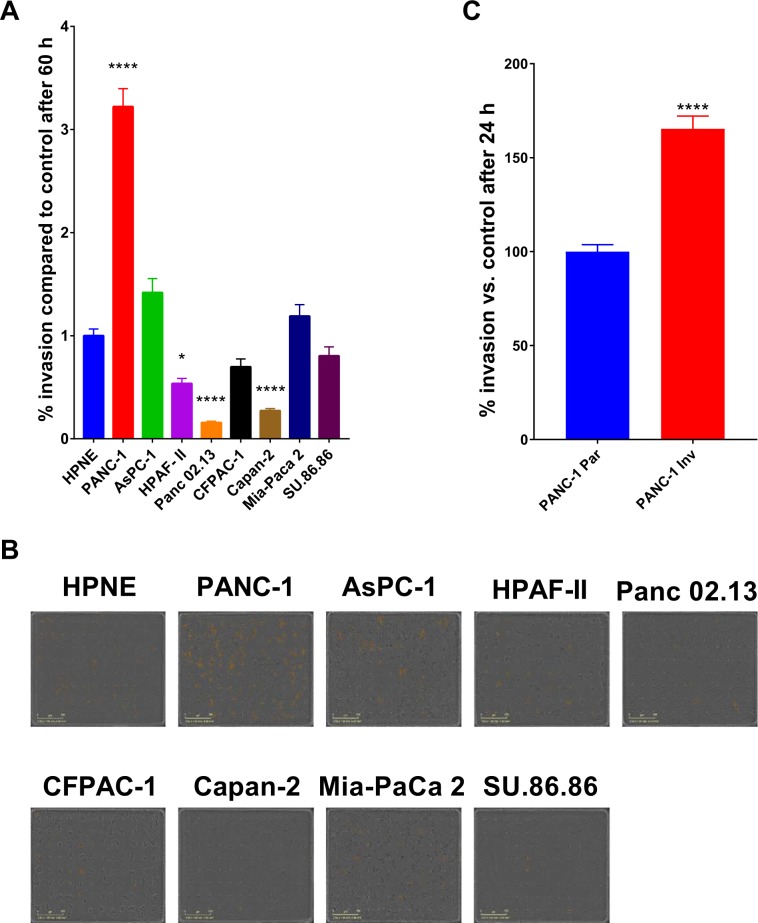
Selection of cell line model for CRISPR screen. (**A**) Eight PDAC cell lines were assessed for invasion and compared to noncancerous HPNE cells (HPNE was used as control; 100%). The graphs represent fold differences in the number of cells that migrated toward a chemoattractant (complete medium). (**B**) PDAC cells subjected to invasion assay. Images are representative of the observed phenotype, and the yellow pseudo-colored cells are those that have invaded the lower chamber of the invasion Boyden Chamber. (**C**) Assessment of the PANC-1 cells (PANC-1 Inv) that were skewed towards being more invasive than the original parental population (PANC-1 Par; set as control at 100%). The data presented are based on three independent experiments, and the *P*-values were determined using ANOVA with Tukey’s HSD test and student’s *t*-test. ****P < 0.0001.

To identify genes that regulate PDAC cell invasion, we used a genome-scale CRISPR approach to screen for single-guide RNAs (sgRNAs) that rendered cells noninvasive in a Boyden Chamber invasion assay. Figure 1 shows a schematic of the workflow of the screening assay. To identify a PDAC cell model for the CRISPR screen, we assessed eight PDAC cell lines for their invasive capacity in comparison with noncancerous HPNE cells. PANC-1 was observed to be the most invasive of the cell lines tested (Fig. 2A,B). Because of the heterogeneity of PANC-1 cells with respect to invasiveness, we passed them through an invasion Boyden Chamber and collected the cells that passed through within 4 days. In this way, we obtained a PANC-1 population that was skewed towards being more invasive (PANC-1 Inv) than the original parental population (PANC-1 Par) (Fig. 2C). The highly invasive PANC-1 cells were then engineered to constitutively express the gene encoding Cas9, enabling us to transduce these cells with an sgRNA library and perform the invasion screening in a pooled format. We transduced the cells with the Brunello CRISPR pooled gRNA library (human sgRNA library Brunello in lentiGuide-Puro; Addgene, catalog # 73178)^10^, and selected for transduced cells with puromycin. The stable cells were then plated in the upper chamber of an invasion assay plate. The cells were allowed to invade for 4 days, and the cells that failed to invade and, thus, remained in the upper chamber were collected for further analysis. The assay was performed as three biological replicates (three independent screens) (Fig. 1). Next-generation sequencing (NGS) was then used to identify sgRNAs from the harvested cells. The NGS was performed on 100-bp amplicons with upwards of 1.5 × 10^8^ reads, of which approximately 90% were mapped to the gRNA library (Supplementary Fig. S1). After performing NGS, we identified enrichment hits by using MAGECK-VISPR software^11,12^. A complete list of hits can be found in the Supplementary Data Set. The hits from the three biological replicates (independent screens) were compiled and ranked. We chose the top 125 hits and re-screened them with pooled siRNAs (knockdown) for each gene and an individual CRISPR gRNAs (knockout) for each gene in PANC-1 cells (Supplementary Fig. S2).

### *MBNL3* and *KANSL2* regulate PANC-1 invasion and migration

**Figure 3:**
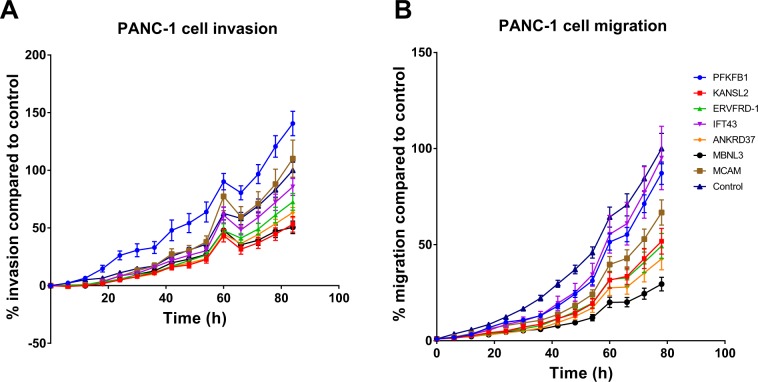
Hit validation for invasion and migration. The top seven common hits from the siRNA confirmatory and CRISPR confirmatory screens were further assessed by CRISPR in PANC-1 cells for (**A**) invasiveness using cell invasion assay, (**B**) migration using cell migration assay. The data presented are based on three independent experiments.

The top seven genes common to the siRNA and CRISPR confirmatory screens were then selected for further analysis by CRISPR-mediated knockout. The knockout of *MBNL3* and *KANSL2* significantly impaired the invasive capacity of PANC-1 cells when compared to the controls (Figs. 3A and S2 [red dot = *MBNL3* knockout, green dot = *KANSL2* knockout]). We also observed that knocking out *MBNL3* and *KANSL2* inhibited cell migration, but the effect of *KANSL2* knockout on migration was milder than that of *MBNL3* knockout (Fig. 3B).

**Figure 4:**
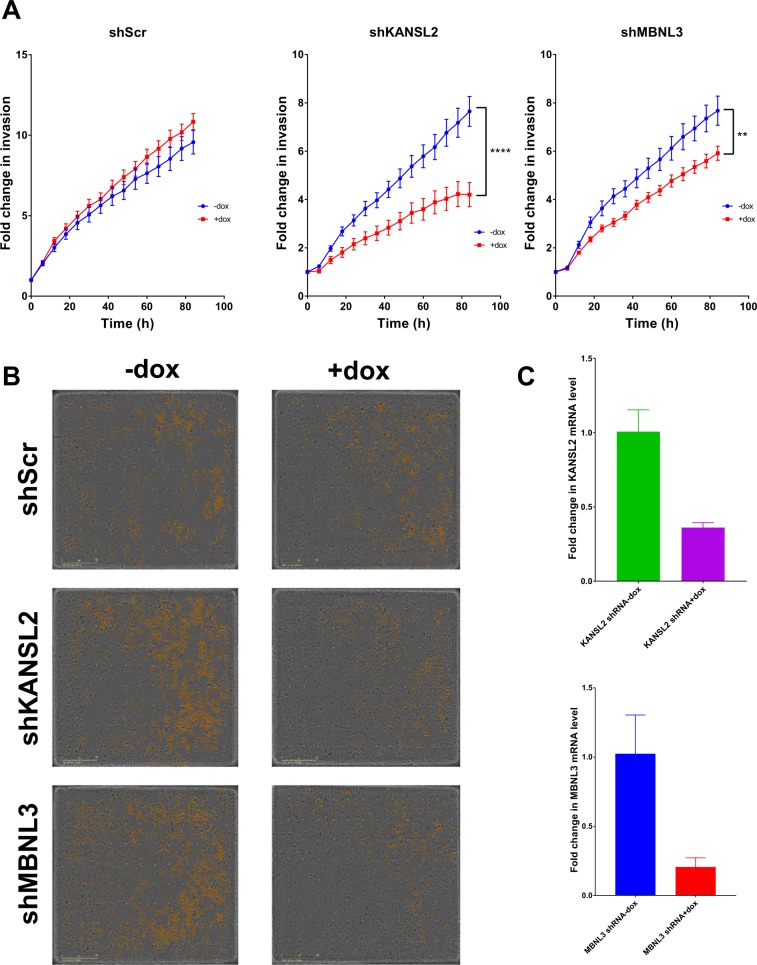
*MBNL3* and *KANSL2* regulate PANC-1 invasion. (**A**) The downregulation of genes giving the strongest response from the hit validation for invasion, *MBNL3* and *KANSL2*, were confirmed by shRNA-mediated knockdown in response to doxycycline versus no treatment. (**B**) Images are representative of the observed phenotype, and the yellow pseudo-colored cells are those that have invaded the lower chamber of the invasion Boyden Chamber. (**C**) The graphs represent the knockdown efficiency of *KANSL2* and *MBNL3* as assessed by the mRNA levels. The data presented are based on three independent experiments, and the *P*-values were determined using ANOVA with Tukey’s HSD **P < 0.01, ****P < 0.0001.

To further confirm our observations on invasiveness, we used a doxycycline-inducible shRNA system targeting *MBNL3* or *KANSL2*. Upon doxycycline treatment, cells expressing *MBNL3* shRNA or *KANSL2* shRNA showed a decrease in their invasiveness (Fig. 4A, middle and right panels; Fig. 4B, middle and lower panels). We observed no change in the invasiveness of the control cells expressing scramble shRNA (shScr) with or without doxycycline (Fig. 4A, left panel; Fig. 4B, top panel). The shRNA knockdown efficiencies of *MBNL3* and *KANSL2* are shown in Fig. 4C. To ensure that our observations were indeed due to an intrinsic change in the invasive capacity of the cells and not a result of a change in their proliferation, we performed a spheroid growth assay. Three growth conditions were tested: cells without doxycycline (no dox), cells treated with doxycycline at the time of seeding (dox added on day 1), and cells that were seeded and allowed to grow for 6 days before doxycycline treatment (dox added on day 6). With each of these three growth conditions, we observed no difference in growth in the scramble control cells, the shKANSL2 cells, or the shMBNL3 cells (Supplementary Fig. S3). This suggests that *MBNL3* and *KANSL2* do indeed regulate invasion in PANC-1 cells.

## Discussion

The rapid metastatic potential of pancreatic cancer is the main reason for treatment being unsuccessful. Typically, patients with either precancerous lesions or early PDAC do not exhibit obvious symptoms. Therefore, the lack of appropriate methods for early diagnosis results in only 20% of PDACs being diagnosed at a stage that is suitable for operation, which contributes to the low survival rate. Usually, by the time of diagnosis, pancreatic cancer has already colonized organs such as the liver, lungs, and spleen, making resection an impossible treatment option. Because pancreatic cancer is so aggressive, the various features of its aggressiveness, such as the evasion of apoptosis, rapid growth, therapeutic resistance, and metastasis, need to be carefully examined in order to develop a better treatment paradigm.

In this study, we aimed to identify genes that play important roles in the metastatic spread of PDAC. We first developed a cell model suitable for studying cell invasion by assembling a panel of eight PDAC cell lines and comparing their invasive capacity. We identified PANC-1 as the most invasive cell line. After further enriching the PANC-1 cells for invasiveness by using a Boyden Chamber, we used this population in a genome-scale CRISPR pooled gRNA library screen to identify genes that, when knocked out, decreased cell invasiveness. The genes so identified were further validated in two separate secondary screens using siRNA or CRISPR, leading to the identification of *MBNL3* and *KANSL2* as robust regulators of PANC-1 cell invasion and migration. Thus, we have established a process to identify genes that regulate cell invasion and that can be further investigated as potential targets for therapeutic intervention.

*MBNL3* gene encodes the muscleblind-like-3 protein, which is primarily known to regulate alternative splicing and recently reported to promote hepatocellular carcinoma^13^. *KANSL2* gene encodes the KAT8 regulatory NSL complex subunit 2 protein, which is a KANSL protein family member belonging to the complex of lysine acetyl-transferase KAT8/MOF-NSL, and recently shown to regulate glioblastoma’s cancer stem-like properties that contribute to tumorigenesis^14^. Our findings that *MBNL3* and *KANSL2* regulate PDAC cell invasion add to the growing knowledge that *MBNL3* and *KANSL2* play important roles in cancer metastasis, and warrant further investigation to define the underlining mechanisms in order to validate them as potential therapeutic targets for PDAC. We chose PANC-1 because it is the most invasive of the 8 PDAC cell lines tested. Further investigation needs to include additional PDAC models such as patient-derived models. We found that MBNL3 and KANSL2 regulate PDAC cell invasion; further investigation needs to define the molecular targets and pathways that MBNL3 and KANSL2 regulate.

## Materials and Methods

### Materials

Fetal bovine serum (FBS) was purchased from HyClone (Logan, UT, catalog number SH30071.03). All cell lines [AsPC-1 (CRL-1682), HPAF-II (CRL-1197), PANC-1 (CRL-1469), MIA PaCa-2 (CRL-1420), SU.86.86 (CRL-1837), Capan-2 (HTB-80), CFPAC-1 (CRL-1918), Panc 02.13 (CRL-2554), and hTERT-HPNE (CRL-4023)] were obtained from the American Type Culture Collection (ATCC, Manassas, VA) and have been authenticated by short tandem repeat (STR) DNA profiling. Puromycin was obtained from Sigma (catalog number P9620), and other cell-culture reagents were obtained from Invitrogen (Carlsbad, CA)^15^. 18S and *KANSL2* and *MBNL3* TaqMan probes were purchased from Thermo Fisher Scientific (Waltham, MA).

### Cell culture

All cell lines were grown in culture media suggested by ATCC and were maintained in a humidified incubator at 37 °C with 5% CO_2_. Cells were routinely verified to be mycoplasma-free by using the MycoProbe Mycoplasma Detection Kit (R&D Systems, Minneapolis, MN, catalog number CUL001B)^15^. PANC-1 cells stably expressing doxycycline-inducible short hairpin (sh) RNA against *KANSL2* (PANC-1 shKANSL2) and *MBNL3* (PANC-1 shMBNL3) were established using lentiviral pLKO.1 plasmids from The RNAi Consortium/Sigma (SHCLND-NM_017822 and SHCLND-NM_133486, respectively). The knockdown of *KANSL2* and *MBNL3* was confirmed by qPCR. Cas9-stable cell lines were made by virally transducing cells with LentiCAS9-Blast (Addgene, Cambridge, MA, catalog # 52962)^12,16^ and selecting with 8 µg/mL of blasticidin for 5 days. Expression was verified by Western blot analysis. Lentiviruses were generated in HEK293T cells (ATCC) in 6-well plates as previously described^12,15^. Briefly, we combined 1 µg of human pLKO vector, 0.75 µg of psPAX2 (a gift from Didier Trono; Addgene plasmid # 12260), and 0.25 µg of pMD2.G (a gift from Dider Trono; Addgene plasmid # 12259) with 5 µL of Lipofectamine 3000 in Opti-MEM for transfection. The transfection medium was replaced with fresh medium after 6 h, and viruses were collected after 48 h. The medium was filtered with a 0.45-µm PES filter to remove cells and debris then frozen at −80 °C. Viral transduction was accomplished by adding 1 mL of virus-containing medium mixed with 3 mL of fresh medium to a 6-cm dish of PANC-1 cells at 40% cellular confluence, with 8 µg/mL Polybrene (Sigma-Aldrich, product number 107689), for 16 h. Puromycin was used to establish and maintain pooled antibiotic-resistant stable cells^15^. For induction, the cells were grown in medium with tetracycline-free FBS before doxycycline was added.

### CRISPR screen

PANC-1 cells stably expressing Cas9 (Addgene catalog # 52962) were transduced (Day 0) with the CRISPR lentiviral pooled gRNA library as previously described (human sgRNA library Brunello in lentiGuide-Puro; Addgene, catalog # 73178)^10,12,16^, and transduced cells were selected with puromycin (2 µg/mL), beginning 72 h (Day 3) after infection; A killing curve for PANC-1 cells was performed to show that puromycin at 2 µg/mL completely killed the cells after 72 hours. The cells were incubated for 8 days (Days 3 to 11) in the presence of puromycin to maximize viral integration and gene expression. The initially transduced PANC-1 cells were split into two equal batches, with a minimum of 3 × 10^7^ cells per replicate, and 3 × 10^7^ cells were cryogenically preserved for subsequent genomic DNA analysis. For the cells selected with 2 µg/mL of puromycin (Days 3 to 11), at day 11, cells were allowed to proliferate in the absence of puromycin for 5 days (Days 11 to 16) and then 1 × 10^8^ cells were collected and frozen for genomic DNA isolation. Next, 1 × 10^8^ cells were plated in each of the invasion assay plates, and the cells were allowed to invade from the upper, serum-deprived compartment into the lower compartment containing complete medium for 4 days (Days 16 to 20). Non-invading cells were collected for genomic DNA isolation. One hundred–cycle single-end sequencing was performed on an Illumina HiSeq. 2500 System. After HiSeq sequencing, the raw FASTQ files were deconvoluted by barcode and trimmed of excess nucleotides in the St. Jude Children’s Research Hospital High Performance Computing Facility. The resulting amplicons were then analyzed with MAGECK-VISPR13 as previously described^11,12^. Based on the actual positive score (pos) from the analysis using MAGECK-VISPR13, each gene was ranked, and assigned a possible numeric value score of 0–4 (to represent the number of positive sgRNAs out of four per gene from the pool of gRNAs). This analysis was performed for all three independent screens. A hit list was then generated to include genes which have an overlap of greater than two positive gRNAs among the three independent screens (1,767 genes); 48 of the 1,767 genes have an overlap of greater than three positive gRNAs. After removing duplicates, a final hit list contains 959 genes. The top 125 genes were further analyzed.

### Genomic DNA isolation and PCR amplification

The DNA isolation and amplification procedures have been described previously^12^. Briefly, genomic DNA was extracted with a QIAamp Blood Maxi Kit (Qiagen, catalog # 51192) according to the manufacturer’s protocol. Using a nested PCR program, we generated barcoded amplicons containing the integrated gRNA sequences. The 10 separate 100-µL redundant reactions contained 5 µg of DNA, HS-Premix Ex Taq (Takara, catalog # RR030A), and 6 µL of each 10 µM solution of primer pairs F1 and R1^12^. The first-round PCR program was as follows: step 1, 95 °C for 1 min; step 2, 95 °C for 30 s; step 3, 55 °C for 30 s; step 4, 72 °C for 30 s, with steps 2–4 being repeated 15 times. A 5-µL aliquot of the reaction mix was then used to seed the second round of PCR, along with HS-Premix Ex Taq, 6 µL of primer R2, and 6 µL of 10 µM F2 primer staggered mixture that contained the Illumina adapters and a barcode to identify the sample after sequencing analysis. The second-round PCR program was as follows: step 1, 95 °C for 1 min; step 2, 95 °C for 30 s; step 3, 63 °C for 30 s; step 4, 72 °C for 30 s; with steps 2–4 being repeated 17 times.

### RNA extraction and quantitative real-time PCR

RNA was extracted using Maxwell simplyRNA Kits and a Maxwell 16 Instrument (Promega) as previously described^15,17^. Briefly, RNA concentrations were measured using a NanoDrop 8000 UV-Vis Spectrophotometer (Thermo Fisher Scientific). All cDNA used in mRNA quantitative real-time PCR (qPCR) analyses was synthesized from extracted RNA by using the SuperScript VILO cDNA Synthesis Kit (Life Technologies) in accordance with the manufacturer’s protocol. The mRNA expression data were generated using Applied Biosystems TaqMan assays (20×) and Fast Advanced Master Mix (Life Technologies, catalog number 4444556). Thermal cycling for qPCR was performed with an Applied Biosystems 7900 HT Fast Real-Time PCR system (Life Technologies) according to the TaqMan Fast protocol. Gene expression was normalized to the housekeeping gene 18S ribosomal RNA (18S), the expression of which did not vary as a function of the experimental conditions. Data are shown as the mRNA fold change (2^−ΔΔCT^) relative to the mRNA level of the corresponding transcript in the control samples as indicated. Each experiment was performed at least three times, and all samples were analyzed in triplicate.

### Cell invasion/migration assays

Real-time cell invasion in response to serum present in the complete medium was measured as the number of cells invading the lower chamber at each time point normalized to the starting number of cells in the upper chamber coated with 50 µg/mL extracellular matrix (ECM) (Sigma). Invasion was monitored using an IncuCyte ZOOM live-cell imaging system (Essen BioScience)^15^. In brief, target genes were knocked down by means of shRNA in cells plated in 10-cm cell culture dishes by treating them with 1 µg/mL doxycycline. Forty-eight hours after induction, cells were trypsinized and plated at a density of 5000 cells/well in IncuCyte ClearView 96-well Chemotaxis Plates. The number of invading cells was assessed every 6 h throughout the experiment. Cells expressing scramble shRNA were used as a negative control. Cell invasion curves were plotted using the number of invading cells normalized to the starting number of cells plated, with the results being presented as the fold change at specified time points for each treatment. For the cell migration, the upper chamber was not coated with ECM. Please note the difference between cell migration and cell invasion: while cell invasion include cell migration, invasive cells are able to move through the ECM. Therefore the cell invasion assay was performed in the presence of ECM, but the cell migration assay was performed in the absence of ECM^18^.

### 3D-Spheroid formation assay

The assay was performed as previously described^12^. Briefly, PANC-1 cells stably transduced with scramble shRNA, or stably expressing shRNA targeting *KANSL2* or *MBNL3*, were seeded into a round-bottom 96-well plate at a density of 300 cells/well. The medium was changed every 3–4 days. Cell viability was measured on day 6 and day 10 by using the CellTiter-Glo 3D Cell Viability Assay (Promega, catalog number G9681) according to the manufacturer’s protocol, with the results being shown in luminescence units.

### Statistical analysis

Statistical analysis was performed as previously described^17^. Briefly, data from at least three independent replicate experiments were pooled and quantitatively analyzed by analysis of variance (ANOVA) plus Tukey’s honest significant difference (HSD) test and by Student’s *t*-test, using GraphPad Prism 7.0 software. *P*-values of less than 0.05 were considered to indicate significance. Results are expressed as the mean ± SE.

## Supplementary information

Supplementary Information.

Supplementary Data Set.

## Data Availability

The authors declare that all data supporting the findings of this study are available within the article and its Supplementary Information.
