# Supplementary material for: KANSL2 and MBNL3 are regulators of pancreatic ductal adenocarcinoma invasion
Source: Sci Rep. 2020 Jan 30;10:1485. doi: 10.1038/s41598-020-58448-y (PMC6992758; doi:10.1038/s41598-020-58448-y)

KANSL2 and _MBNL3_ are regulators of pancreatic ductal adenocarcinoma invasion

Peter O. Oladimeji[1], Jesse Bakke[1,2], William C. Wright[1,3] & Taosheng Chen[1,3*]

[1]Department of Chemical Biology and Therapeutics, St. Jude Children's Research Hospital, Memphis, Tennessee 38105, USA.

[2]Current address: Department of Foundational Science, Central Michigan University, Mt. Pleasant, Michigan 48859, USA

[3]Integrated Biomedical Sciences Program, University of Tennessee Health Science Center, Memphis, Tennessee 38163, USA.

*Correspondence should be addressed to Taosheng Chen, Department of Chemical Biology and Therapeutics, MS 1000, St. Jude Children's Research Hospital, 262 Danny Thomas Place, Memphis, TN 38105, USA. Tel: (901) 595-5937; Fax: (901) 595-5715; email: taosheng.chen@stjude.org

Supplementary Figure S1. Next-generation sequencing and enrichment results. (A) Total reads, both mapped and unmapped. (B) Candidate genes were identified after an enrichment CRISPR screen. (C) Gini index (a measure of inequality, with 1 being the most unequal) of the starting cell population and the three replicates of the enrichment screen.

Supplementary Figure S2. Confirmatory screens of top hits. Waterfall plot showing the distribution of the top 125 genes selected from the primary screen in a confirmatory CRISPR screen (upper panel) and a confirmatory siRNA screen (lower panel). The red dot is the position of *MBNL3* and the green dot is the position of *KANSL2* after ranking.

Supplementary Figure S3. Results of spheroid growth assay. The fold change in proliferation of PANC-1 shScr, PANC-1 shKANSL2, and PANC-1 shMBNL3 cells in spheroids treated with or without doxycycline as described in the Results and Methods sections. The data presented are based on three independent experiments.

Supplementary Data Set. Hit list from three independent CRISPR screenings. The first three columns list the ranked genes from each independent CRISPR screen (Rep 1, Rep 2, and Rep 3, respectively). The fourth and fifth columns list the genes having an overlap of at least two positive gRNAs (“pos/goodsgrna”; 1,767 genes) or three positive gRNAs (48 genes) among the three independent CRISPR screens, respectively. Column 6 lists the final hits (959) after removing duplicated genes, with the top 125 hits highlighted in yellow.

Supplementary Fig S1

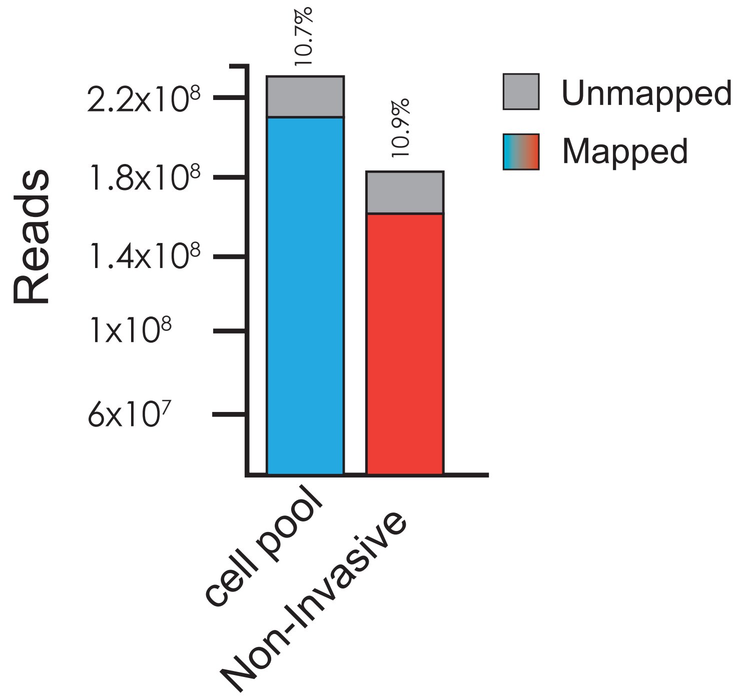

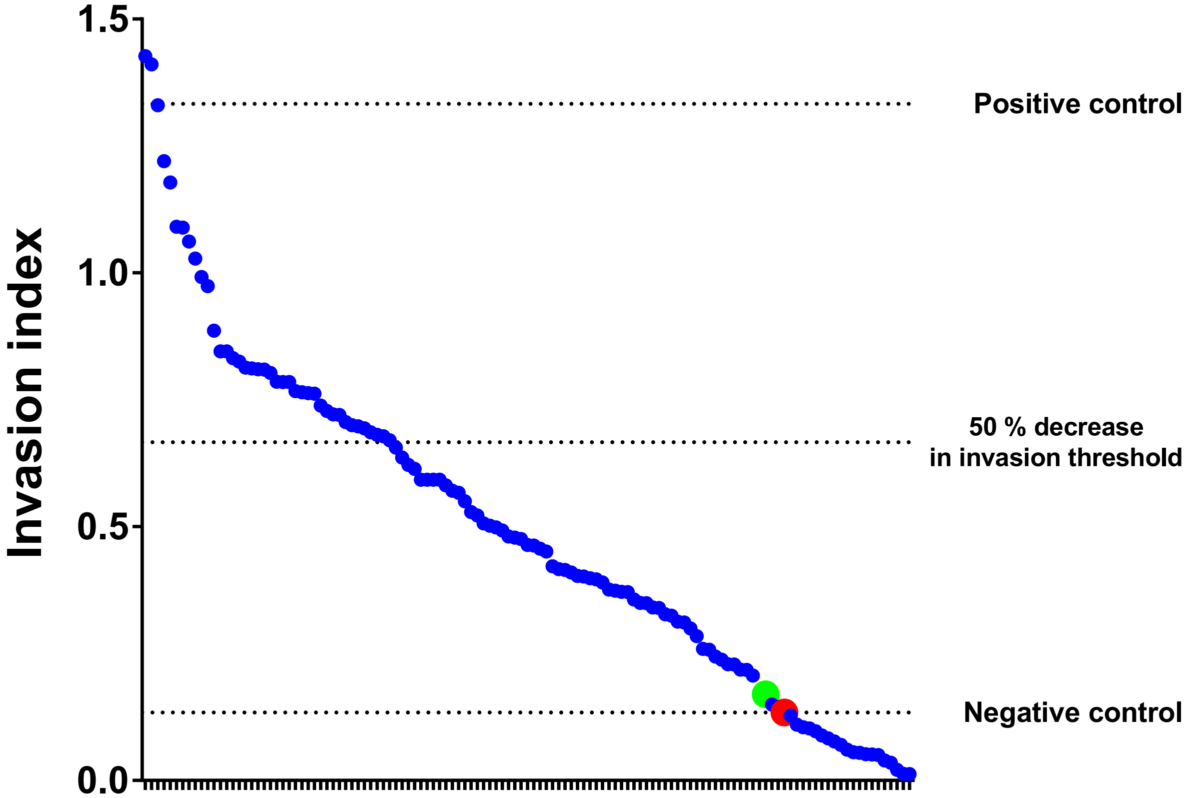
1.5
1.0
0.5
0.0
Invasion index
Positive control
50 % decrease
in invasion threshold
Negative control

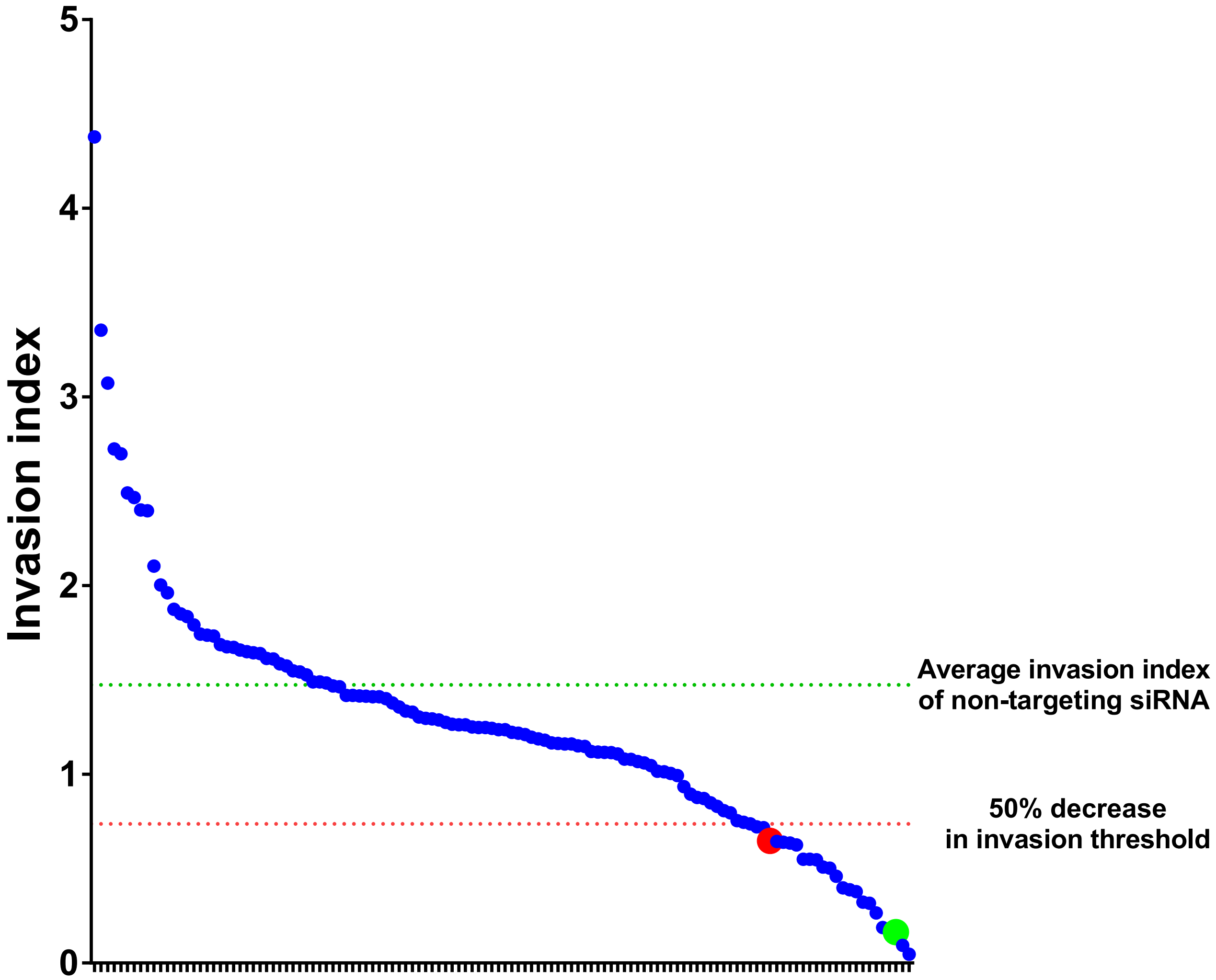
5
4
3
2
1
0
Invasion index
Average invasion index
of non-targeting siRNA
50% decrease
in invasion threshold

Supplementary Fig S3

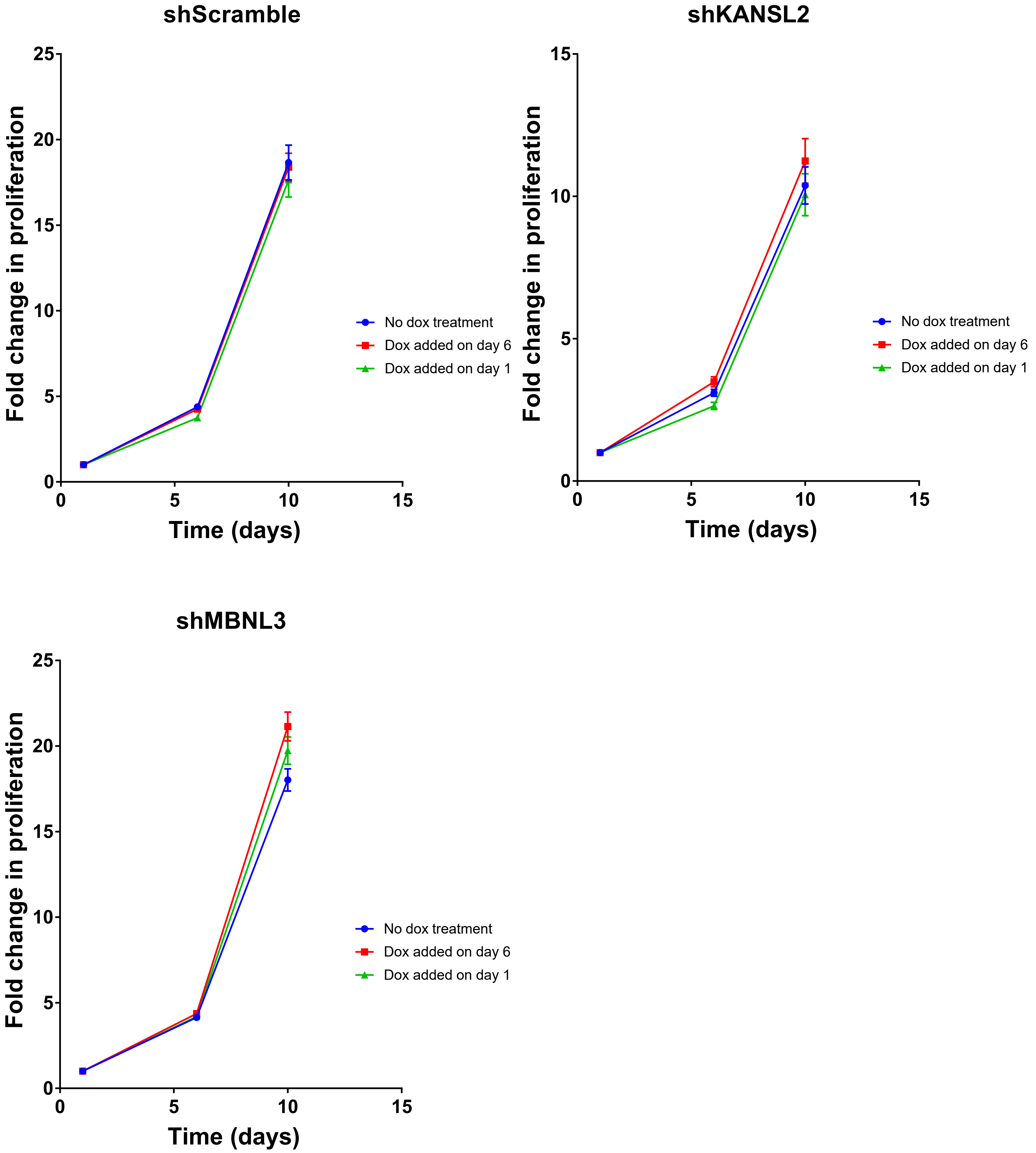

Supplement: Supplementary file 1 — Supplementary Information. [file 41598_2020_58448_MOESM1_ESM.pdf]
